# The use and misuse of health research by parliamentary politicians during the development of a national smokefree law

**DOI:** 10.1186/1743-8462-4-24

**Published:** 2007-12-06

**Authors:** George Thomson, Nick Wilson, Philippa Howden-Chapman

**Affiliations:** 1 University of Otago, Wellington, New Zealand

## Abstract

**Background:**

We investigated the ways in which research evidence about the health effects from secondhand smoke (SHS) and smokefree policies was publicly used or regarded by New Zealand parliamentary politicians, during efforts to strengthen a smokefree law (ie, from 1997 to 2005).

**Methods:**

A documentary case study used published and unpublished material recording the use of research evidence by politicians. The material was collected for the period 1997–2005 from the parliamentary record, media and other databases. Additional searches were made to provide context for the politicians who used research.

**Results:**

Major themes identified included: (a) the employment of local estimates of SHS mortality, (b) linking specific health effects (eg, cancer) to SHS exposure, (c) a focus on the use of research relevant to bar workers, and (d) the use of research to downgrade the health effects, and attacks on the credibility of research showing health effects from SHS. Almost half of the 21 Members of Parliament (MPs), who spoke in parliament about SHS research during 2000–2005, denied or were sceptical about SHS harm. At least five MPs used tobacco industry funded or disseminated versions of research. There was some indirect evidence that the degree of exposure to the health sector, or the tobacco industry and its allies, may have been factors in the use by MPs of the research.

**Policy implications:**

The willingness of some of this group of politicians to adopt tobacco industry arguments suggests possible options within health promotion. These include the better enforcement of consumer protection laws (preventing deceptive information by the tobacco industry and its allies), and the adoption of an increased focus on tobacco industry behaviour within tobacco control efforts. These moves may have beneficial effects for the use of research in public health policymaking. The strengthening by the health sector of its advocacy capacity and effectiveness may also be a crucial step in the better use of research by politicians in the policymaking process.

## Background

Despite the critical nature of the use of law to advance public health, relatively little is known about the use of research evidence in the policymaking process for evidence-based health-protecting laws. There is some literature which suggests that politicians have limited time and incentives to absorb research details, and often need to see an issue at a personal level [1]. The reputation and credibility of research providers is important to politicians, as is the local relevance [2]. Research may be most influential in framing policy debates [3,4]. The extent of the acceptance and use of research may depend on the context of the 'prevailing narratives', the degree to which there are political motives for which to contest the research, the attractiveness of the research in solving community and political problems, and the clarity of the communication of the research [2]. That communication requires the systematic promotion of interaction between researchers and policymakers,[5,6] in which professional health research communication and advocacy can be vital [7,8].

However, evidence from Canada indicates that accurate knowledge by legislators of harm to health from smoking is related to their declared support for tobacco control policies [9]. There is some population evidence that the depth of knowledge and the emotional value of the information about smoking's ill-effects is important in deciding consequent decisions [10,11]. For the causes of chronic diseases in general, there is some evidence that relevant evidence does not effectively reach policymakers [12].

Given the critical importance of evidence-based policies for advancing tobacco control and other aspects of population health,[12,13] the considerable obstacles to effectively translating research results to policy,[1] and the need to better understand upstream obstacles,[14] we sought to further explore the intersections between research and policy development. In particular, we examined the public statements on health research by New Zealand parliamentary politicians involved for and against smokefree policies, during 1997–2005.

International and New Zealand reviews published between 1986 and 2002 consistently found significant health risks from SHS (eg,[15,16]). Against this research consensus, the international tobacco industry has attempted to create doubt about the strength of this evidence in New Zealand and elsewhere from at least 1981 [17-26]. The industry's efforts included funding of researchers, whose research then appears far more likely to conclude that SHS is not harmful [27]. The New Zealand context includes the recognition by large majorities of the public, for over 20 years, that there are at least some health risks from SHS. Since 1999 this proportion has been 90% or over,[28] although this recognition may not include the extent of the serious harm and deaths resulting from SHS [29-31].

The New Zealand Smoke-free Environments Act from 1990 required offices, shops and some other workplaces to be smokefree, but by 1996 about 20% of workers were still exposed to SHS at work [32]. In 1999, a Private Member's Bill was introduced, proposing extended smokefree workplaces,[33] and it progressed to the Parliamentary Select Committee in 2000. It was strengthened and passed in December 2003, with most of the measures implemented in December 2004. Three out of the six political parties in the Parliament (National, United Future, New Zealand First) did not impose a party policy for the vote, and allowed MPs to vote as they wished. In mid 2005, a Private Member's Bill was introduced by an opposition MP to reverse some of the smokefree measures, but was defeated. New Zealand has a unicameral, Westminster parliamentary system, where members are elected by mixed-member proportional representation.

The use during 1997–2005 by New Zealand politicians, of health research evidence about SHS, occurred against a background of disinformation by the tobacco industry and its allies. This included statements to the Health Select Committee of Parliament in November 2002,[34] and by the Hospitality Association of New Zealand, a major opponent up to 2004 of the efforts to protect workers from SHS [35,36]. The statements expressed doubt or denial of the increased risk of cancer and heart disease from SHS exposure.

## Methods

### Definition

Research is defined for this article as the systematic collection and analysis of data.

### Data collection

An intrinsic documentary case study was used, in this case, the study of a group over a limited period, so as to better understand them and the policymaking process [37-39]. Published and unpublished material recording the use of research evidence by politicians (about the health effects from secondhand smoke (SHS) and smokefree policies) was collected for the period 1997–2005. This included: media comments; official statements and other official documents; and parliamentary speeches, questions and replies. The period 1997–2005 was selected because it covered the genesis, parliamentary process, and initial year of implementation of the main part of the Smoke-free Environments Amendment Act (SFEAA).

A database for the New Zealand *Parliamentary Debates *was searched for the period 2000–2005, using the search word 'smoke' [40]. The debates on the proposed law, recorded in the *Parliamentary Debates *for the 11 days in which the Bill was debated during 2000–2003, was read and examined for uses of research. The Factiva database for news media[41] was searched for the New Zealand region in the period 1997–2005, using the search word 'smokefree' with either 'parliament' 'minister' or 'government'. Databases for ministerial statements, releases and speeches[42,43] were searched for the years 1997–2005, using the search words 'smoke' and 'tobacco'.

To provide context, searches were made in the Parliamentary services database [44], the Factiva database, tobacco industry document databases, and in the literature on New Zealand politics.

### Data analysis

The data analysis was initially formed by the research question 'what were the ways in which research evidence was used by the politicians'. An iterative and inductive process was used to search for patterns and themes, generally using constant comparative analysis [45-48]. The themes were adapted during the reading and rereading of the material, and the types of themes were discussed by the three investigators and modified.

To help guard against bias, all references during 2000–2005 in the *Parliamentary Debates *(about the health effects from secondhand smoke (SHS) and smokefree policies) that were found were used in the analysis. Efforts were made to ensure that evidence from other sources (eg, media, official statements) was equally sourced from both those supporting and rejecting tobacco industry interpretations. Explicit consideration was given to data that went against the main thrust of the available material, and efforts were made to ensure that opposing viewpoints from the data were given when available [48].

## Results

We found 89 documents recording the use of research evidence, during 1997–2005, by New Zealand politicians involved for and against smokefree policies. Sixty eight of these documents (76%) were from the years 2000, 2001 and 2003, the years when the smokefree places legislation received the most media and parliamentary attention. Forty six of the documents were speeches or groups of speeches in Parliament. Within this material, three major areas were explored: (i) the use of research evidence about the health effects from SHS, (ii) who used the research, and (iii) some of the ways research was used to try and affect policy.

### The use of research evidence about the health effects from SHS

Major themes identified in the use of SHS health effects research included: (a) the use of local estimates of SHS mortality; (b) linking specific health effects (eg, cancer) to SHS exposure; (c) a focus on the use of research relevant to bar workers; and (d) the use of research to downplay the health effects, and attacks on the credibility of research showing health effects from SHS. In addition, politicians used research about the indirect health effects of smokefree policies, including the impact on youth smoking uptake [49].

### The use of the Woodward and Laugesen research of 2000

The period 2000–2005 saw the repeated employment of the research by Woodward and Laugesen on estimates of mortality in New Zealand from SHS (first published as a report for government, then as a journal article) [50,51]. In their research, overseas data for disease risks from SHS was matched with New Zealand data for SHS exposure, and the rates of deaths in New Zealand by tobacco-attributable causes. The derived estimate in the report was 388 deaths per year, and in the article 347 deaths per year, in both cases giving the 'plausible range'. The authors noted:

'Attributable risk estimates provide an indication for policy makers and health educators of the magnitude of a health problem; they are not precise predictions'[51].

The Minister of Health stated that:

'The new research indicates some 388 deaths a year are attributable to second-hand smoke,' that the research: 'confirms the need for stronger measures to protect people from exposure to second-hand smoke' and noted that the number killed was: 'about three quarters the number of people killed each year on New Zealand roads'[52].

Ministers used the mortality research in at least five more official statements outside of Parliament, supporting the smokefree legislation change [53-57]. In Parliament, five MPs used the mortality figures to support smokefree policies, in eight speeches or replies to questions, [58-65] as would the Health Select Committee in its report to Parliament on the draft legislation [66].

### Using research on specific health effects and the effects for bar workers

After the use of mortality estimates, the most common use of research was to mention the specific health effects of SHS exposure. Cancer, heart disease, and stroke were the diseases normally cited,[49,67-69] but sudden infant death syndrome (SIDS), asthma, middle ear disease and respiratory infections were also often mentioned [49,64,67,68,70,71]. In addition, politicians used research evidence to argue that smokefree policies would lead to better health outcomes [72].

Statements by politicians included references to research on the health risks to bar workers from SHS, and on the health improvements for bar workers due to smokefree policies. The statements noted the higher SHS exposure of bar workers compared to office workers, and higher lung cancer risks for bar workers compared to the general population [61,67,73,74].

### The scepticism about and rejection of health evidence about SHS

In doubting or rejecting the health evidence, the arguments included those about the research methods used, the alleged qualification of SHS risks by the World Health Organization (WHO), arguments about 'more dangerous' risks than SHS, and about an alternate 'common sense' approach. During the Parliamentary debates during 2003 and 2005 on smokefree policies, at least nine MPs from four different parliamentary parties cast doubt on the evidence of harm to health from SHS – Doug Woolerton and Dail Jones (New Zealand First), David Carter and Richard Worth (National), Heather Roy and Deborah Coddington (ACT) and Peter Dunne, Marc Alexander and Paul Adams (United Future) [75-85]. Another MP (Winston Peters) doubted the expertise of those using the research [86]. Bill English MP minimised the size of the risk [87]. In addition, Dr Lynda Scott MP indicated some reservations about the quality of research evidence, but not about the overall message:

'some of the evidence has been overestimated, but there is no doubt that second-hand smoke does affect people'[88].

A number of MPs made efforts to counter this scepticism or denial [71,89]. Sue Kedgely MP characterised the scepticism or denial by MPs as: 'singing the tobacco industry tune, that somehow the research is inconclusive'[59].

### Attacks on the SHS mortality estimates

During August 2003 – July 2005 there was a substantial effort by some parliamentary opponents of the legislation to cast doubt on the Woodward and Laugesen estimates. At least five MPs made parliamentary speeches in which varying arguments were made against the use of the estimates. The opponents disagreed with the idea of estimates being derived from data for disease risks, SHS exposure, and for rates and numbers of deaths by cause. They asked for more concrete evidence. David Carter MP asked that the legislation supporters:

'table the 400 death certificates that have been filed and state that those people died of secondhand smoke' and said: 'that member will not be able to table those death certificates. .... Not one, because they do not exist. What we see here is a huge manufacturing and embellishment of the story about the effect of second-hand smoke on people's health'[75].

Another concern with the methods used in the mortality estimate was that there were some unknown variables involved. An MP (Dail Jones) expressed concerns about the methods, including the use of previously published research to establish the disease risks from SHS. He appeared to expect Woodward and Laugesen to establish all the data from basic research:

'When people do a research they do it on actual cases. They do not go searching the literature, which other people have written, so that they can write about it'[76].

Doug Woolerton MP was also concerned about the methods used for the mortality estimates. He stated that 'it is said that thousands of people are dying in this country through second-hand smoke',[77] that for the official estimates any death of an ex-smoker is blamed on smoking, and that such an ex-smoker 'apparently becomes a victim of secondhand smoke'[77]. Another MP (Heather Roy) described the methods used for the mortality estimates as a 'bit of arithmetic, and some heroic assumptions'[78].

Jones further suggested that the work by Woodward/Laugesen had been 'discredited by Dr Proctor'[90]. Dr Chris Proctor, director of science and regulation for British American Tobacco (BAT), had visited New Zealand in 2001 and 2002,[34,91] and had been reported as saying that the SHS mortality figures 'could not be proven'[92]. An industry consultant, Peter Lee, had reviewed the Woodward/Laugesen report for BAT in 2000 [93].

In the arguments about SHS mortality, the statements that WHO had heavily qualified or denied the risks of SHS were part of a wider use of this tactic by parliamentary and other opponents of smokefree laws (detailed further below). The statements appeared to be based on the idea that the 1998 study at the International Agency for Research on Cancer (IARC) on SHS cancer risks had *not *shown that there were risks [94]. In fact the study was consistent with other studies in showing that there *were *such risks. The international tobacco industry's efforts in creating and spreading this idea have been described [95]. Deborah Coddington MP stated in 2003 that:

'The World Health Organization has come out and said that 400 people dying each year from second-hand smoke is a very dodgy statistic'[79].

She repeated the idea that the estimate was 'dodgy' in 2005 [80]. A further MP (Paul Adams) appeared to want to put the Woodward and Laugesen SHS mortality estimates into a 'common sense' perspective. This perspective included Adam's anecdote about his father who had apparently suffered no adverse consequences of smoking [81].

### Other attacks on research on the health risks of SHS

Beyond the attacks that related to the SHS mortality estimates, other arguments on the health risks included further ones about the alleged qualification by the WHO on SHS risks. There were also arguments about other research that might suggest that the risks from SHS were not significant, and about 'more dangerous' risks than SHS. In 2002, the then leader of the Parliamentary Opposition, Bill English (an ex-Minister of Health), justified his support for allowing smoking in bars by describing the consequent danger to staff and customers as 'a small health risk'[87].

The further statements that the WHO had heavily qualified or denied the risks of SHS included those by MPs Jones and Roy. The latter stated that:

'the effects of second-hand smoke ...are inconclusive. ...a controversial World Health Organization study ... found ... that non-smokers married to, working with, or raised with smokers were no more at risk of lung cancer than anyone else'[78].

This view was reiterated in her minority view in the March 2003 Select Committee Report to Parliament [66]. Dail Jones MP also misquoted,[90] the WHO Report of 2002 [96]. Contrary to his suggestion that it showed 'no supporting evidence' for harm from SHS, in the section referred to by Jones (p.66 box 4.1) the report summarises the respiratory, SIDS, heart, and cancer effects of SHS.

Another MP, Richard Worth, referred to the Enstrom and Kabat article of 2003 about SHS mortality [97]. This was a tobacco-industry funded article, where the use and interpretation of the data has been highly disputed [98-104]. Worth said the study:

'has found no significant evidence that second-hand smoke causes lung cancer or heart disease.' He went on to quote the article 'The association between passive smoke, and coronary heart disease and lung cancer, may be considerably weaker than generally believed'[82].

Later, Worth stated that, 'I do not accept the health arguments that lie behind this Bill'[82]. Peter Dunne MP also appears to have cited the 2003 Enstrom and Kabat article while doubting SHS effects [83].

A final tactic by those sceptical about the validity or relevance of evidence about SHS was to compare other risks with SHS exposure. Jones stated that:

'Custard is more dangerous than second-hand smoke. ... milk ...is worse than second-hand smoke. ... Baldness is worse than second-hand smoke. ... Those are the statistics'[90].

Doug Woolerton MP stated:

'This Bill is not based on fact. If it was based on health ...then we would ban motorcars immediately. ... [and home fires] ... Worldwide those things are the biggest contaminants of our environment'[84].

Finally, in 2005 Marc Alexander MP argued that if there was a risk from SHS, why did Parliament not legislate against other air pollutants?[85].

### Who used the research?

During 2000–2005, there were at least 21 MPs speaking in Parliament who used research or attacks on research to argue about smokefree policies. This was out of 48 speakers in the parliamentary debates and questions around the SFEAA, and out of 150 MPs altogether in the two successive parliaments. Of the 21 MPs, 11 were in support of, and 10 were against, the smokefree policies (Table 1). In addition, during 1997–2005 there were at least three MPs (Delamere, English and Tamihere) who only made statements about the research outside of Parliament.

**Table 1:** Political party affiliations of MPs using or attacking SHS research (in parliamentary speeches or questions 2000–2005) and MP voting patterns in 2003 on extending smokefree policies (for MPs on the Health Select Committee during 2000–2003)

	**MPs using or attacking SHS research**			**Voting pattern by the MPs on the Health Select Committee**^b^		
**Party**^a^	**For**	**Against**	**Total**	**For**	**Against**	**Total**
Greens	Kedgley	0	1	Kedgley	0	1
Labour	Chadwick, Dyson, Hughes, King, O'Connor, Turia^c^	0	6	Chadwick, Hartley, Hereora, Mahuta, O'Connor, Yates	0	6
NZ First	Paraone	Jones, Peters, Woolerton	4	Paraone	0	1
United Future	Turner	Adams, Dunne, Alexander	4	Turner	0	1
National	Hutchinson, Scott	Carter, Worth	4	Hutchinson, Scott	Collins, Sowry	4
ACT	0	Coddington, Roy	2	0	Roy	1

**Total**	11	10	21	11	3	14

a – Descending order approximately represents the "left to right" political gradient of the political parties.

b – Phillida Bunkle was a member in 2000–2002, but was not re-elected in 2002.

c – Tariana Turia spoke as a Labour Party MP in 2001, but later became a Maori Party MP.

The background of the MPs who used or attacked the research varied in the exposure to possible influences, such as health experience or closeness to the tobacco industry and its allies. All four MPs from the National, New Zealand First and United parties who voted for the smokefree law in 2003 (Paraone, Turner, Hutchinson, Scott) had been on the Health Select Committee. Hutchinson and Scott were medical practitioners, and Paraone was listed as the director of a 'Maori health company'[105]. Turner has been a member of a hospital chaplains' committee,[105] although she has been reported as saying 'I have never worked in the health sector. So coming up to speed on health issues and health legislation is a huge challenge'[106].

Other MPs who spoke about the research also had health backgrounds. Chadwick has been a nurse, midwife and health sector manager. King, Dyson, and Turia had health-related Ministerial positions from 1999 onwards, and O'Connor and Kedgley had been on the Health Select Committee [61,105]. Hughes had been an assistant to the chairperson of the Health Select Committee during 1999–2002. Roy, the one Health Committee member who doubted the health effects of SHS in Parliament, had been a physiotherapist [105].

Worth was the chairman of the law firm Simpson Grierson from 1986 to 1999.[105] During 1986–1999, this firm worked for the Tobacco Institute of New Zealand and a number of tobacco companies [107-110]. Dunne has consistently voted against tobacco control legislation, and his relationship with the tobacco industry has included a payment to him [111]. Carter has been a hotelier,[105] and had maintained some links with the Hospitality Association [112]. He was reported to be one of two National Party MPs in a meeting with seven BAT managers in 2004 [113].

Some of the MPs using or attacking research appear to have had ideological stances on smoking. Jones was reported to be an asthmatic who 'within half an hour of being exposed to cigarette smoke ... would have an asthmatic attack' but who opposed strengthened smokefree policies 'because it restricted a person's freedom of choice'[114]. Woolerton was reported in 2000 as having personally defied a smoking ban in his parliamentary office for over six months [115]. He was described in 2004 as remaining 'a passionate advocate of smokers' rights' even after giving up smoking [116].

### How was research evidence used to try to affect policy?

Some indication was found of four ways in which research evidence was used to try and affect policy. They were: (i) by helping get policy change onto the political agenda, (ii) by the exposure of politicians to health professionals using research, (iii) by repeated exposure of politicians to a range of evidence, and (iv) by positioning the research as reliable, 'expert', or 'accepted', and therefore 'preferable' compared to other types of evidence.

An example of the use of research to get policy change onto the political agenda occurred in 1997, at a time when the idea of extending smokefree places policies was being revived. The new Associate Health Minister, Tuariki Delamere, responded to articles about the health risks of SHS in the *British Medical Journal*,[117,118] by suggesting that smokefree laws for 'restaurants, shopping centres, bars and indoor public areas should all be looked at'[119].

The exposure of politicians to health professionals, who combined research evidence and personal testimony, may have been effective in influencing statements and consequent policy. This possibility occurred most clearly in Health Select Committee hearings on the proposed law. Except for three MPs (Roy, Collins and Sowry) all 14 of the MPs who sat on either of the two successive Health Committees in 2001–2003 listening to evidence on the Bill (*and *were MPs in 2003), voted for the Bill (Table 1). Pita Paraone gave an indication of the combined effect, for some, of research and personal submissions.

'It is one thing to be told all these statistics; it is another thing to witness them in reality. Health professionals do so on a daily basis, and that is why one would be hard-pressed to find a health worker who is not in favour of limiting the opportunities for smoking and exposure to smoke'[74].

Another MP on the Health Select Committee (Turner) also indicated that the combination of research evidence and health professional testimony was persuasive. Her words also illustrate a precautionary approach to considering the arguments.

'Regardless of the suspicion that people hang over the evidence of second-hand smoke being detrimental to people's health, there is sufficient evidence and backing from medical professions to suggest that those of us with no medical background need to take heed, and I plan to do that'[120].

The Associate Minister in change of tobacco control in late 2003, O'Connor, indicated that after initial scepticism, repeated exposure to research evidence was convincing:

'having sat on the Health Committee for a year .... listening to submissions from the top to the bottom of this country, and hearing the evidence, I can say that it is undeniable and irrefutable that second-hand smoke kills people in New Zealand'[61].

A further MP (Chadwick) used the experience in the Select Committee to argue that this gave a greater breadth of knowledge than was available to New Zealand MPs in general:

'those of us on the Health Committee – those of us who had an open mind – heard all the science and all the reports from around the world, and that gave us the very clear steer that we [should have] a total smoke-free environment'[71].

Politician users of research positioned the research as reliable, 'expert', or 'accepted', or conversely attacked users of research as non-expert or unable to understand research. Sue Kedgley MP stated as part of an argument:

' If the experts are correct, and we can only assume they are ....'[58] In another speech she stated: 'the truth is that there is, despite all the efforts to discredit it, overwhelming medical and scientific consensus that second-hand smoke is the leading cause of death and disease'[59].

Hutchinson defended the source of some of the research used in the debates, Dr Laugesen, as someone who had:

'worked extremely hard to ensure that the information we have in New Zealand is of an absolutely best-practice, world-class quality, [he had] been published in international, peer-review papers, and [he had] data that is very solid indeed'[89].

In attacking the idea of smokefree policies, another MP (Peters) challenged the use by MPs of research evidence because they weren't scientists or 'experts'[86]' Conversely, Health Minister King portrayed an MP attacking smokefree policies, Jones, as someone who could not understand research, as he had:

'been asleep for 20 years, because he does not know that evidence and science today can link tobacco consumption with cancer, and with other illnesses in New Zealand and around the world.'

Jones: 'Where is the proof?'

King: 'A man who could deny that is a man who does not believe in science'[62].

## Discussion

### Main results

The results suggest two main thematic areas: (i) what research is effective for political use and why, and (ii) the issues that arise from tobacco industry efforts to obscure tobacco-related evidence.

### What research works in politics and why

The repeated use of and attacks on the Woodward/Laugesen SHS mortality estimates may indicate the importance of this type of evidence. Research giving a quantified health impact, particularly numbers of deaths, appears to be attractive to politicians. While based on disease risk data from other countries, the research was a way of making the New Zealand health impacts more concrete, and allowing comparisons with other causes of preventable mortality. Estimates of the numbers of deaths make previously little-noticed deaths from SHS into a problem comparable to road crashes. Similarly, research showing specific diseases caused by SHS, and specific effects on bar staff, also enabled politicians to make the harmful impacts of SHS more concrete.

On the other hand, the figures were seen by some politicians to be vulnerable or inadequate, because of the epidemiologic basis for them. Few people can personally identify any victims of SHS, whereas road crash victims are named daily by the media. In particular, for the large proportion of the SHS deaths due to cardiovascular disease, the victims are not readily identifiable.

Research evidence of SHS health effects is further important to politicians, because it helps them support government intervention to protect those who can be seen to be involuntarily affected. It helps in defending against 'nanny-state' accusations. The wide use of research results about specific health effects indicates that while *some *sort of harm from SHS may be taken for granted by the large majority of New Zealand adults, politicians felt the need to detail some of the *specifics *of the harm. Their view is supported by New Zealand research that indicates that the public knowledge of the harm from SHS may not be very deep [29-31].

### What tactics and factors work for the tobacco industry

The denial of significant SHS risks, and the attacks by a group of MPs on the research showing significant risks, suggests at least two things. Firstly, the tobacco industry and its allies appear to have been successful in persuading a proportion of national level politicians that there was significant doubt about SHS health risks. Secondly, that a proportion of the politicians can be willing to both publicly disagree with the beliefs of 90% or more of the adult population,[28] about research results of public health importance (that there are health risks from SHS), *and *to not take a precautionary approach to protect health. We do not suggest that public opinion is a necessary guide to good decision making, but it is of concern from a public health perspective that these politicians did not take this precautionary approach. The causes of the willingness of these politicians to go against public opinion in this case are a question for further research.

Almost half the MPs, who during 2000–2005 spoke in parliament about SHS research, denied or were sceptical about SHS harm. There was a general theme, in the attacks on the research on SHS health effects, that echoed tobacco industry statements. Why has the tobacco industry been successful in this way? Well organised and funded industries have many effective techniques for affecting politicians' ideas [121-125]. The tobacco industry's efforts in pushing their story on the 1998 IARC report, and in funding and disseminating the Enstrom and Kabat research, appear to have paid dividends in New Zealand, with at least five MPs using that story and research.

Other possible reasons for scepticism and denial include possible repeated exposure to the tobacco industry or their allies, being a smoker, and personal or party ideology. The 'self-exempting' beliefs (varied forms of denial) of some smokers about the health risks of smokers may extend to their stance on smokefree policies [126-128]. Political ideology (including beliefs about the role of government) may help determine politicians' attitudes to research[9] (eg, as possibly in the case of Jones). A further possible reason may have been the perception by politicians that few of the public might become aware of the MPs' statements during parliamentary debates.

However, the extent of the industry's success should not be overstated. It may be that the proportion of New Zealand MPs doubting SHS harm is similar to the proportion in the general population.

Finally, for both those accepting and those sceptical of research about SHS, a crucial factor in their stance may have been the degree to which the research was successfully presented as a 'story' or series of stories. In this context, a story goes beyond research findings to become persuasive, through dramatic structure, timing and presentation [129,130]. The story needs to highlight and/or solve a problem [131]. The 'stories' about SHS, that were effective for different groups of MPs, clearly differed.

### Comments on methods, limitations and further research

The use of electronic databases meant that a large number of statements by politicians could be readily accessed. Such documentary case study methods can provide a means of studying some aspects of health policy that does not require intensive resources. The use of documents that were close to first person public statements, or were recording such statements, limited the possible distortion of the words and opinions of the politicians concerned.

However, even such statements may need to be treated with scepticism. Parliamentary speeches may record 'heat of the moment' opinions, and official statements may reflect a degree of group consensus that is different from candid statements from individuals.

The small sample of politicians who spoke about research means that the results cannot be readily generalised to New Zealand politicians as a group. However, while this sample was only 21 out of the 48 (44%) who spoke in parliament about the smokefree policy proposals, they may have been more representative of those politicians who were in key policy development roles within their parties on smokefree policy issues. The early stage of the use of a mixed-member proportional system for New Zealand parliamentary elections (used since 1996) meant that the range and membership of the parties in parliament during 1997–2005 was in constant flux, and thus not necessarily representative of a longer-term situation with this type of political system.

Furthermore, the description and selection of themes in this type of research will to some extent be formed by the backgrounds of the researchers involved. For instance, an analysis of the data by those outside the health sector, or who have not been involved in tobacco control research to the extent of the authors' involvement (see 'Competing interests' below), might produce significantly different themes. The prospects of any such 'neutrality' on tobacco control policy, and all public policy research, are debated [132-134].

In-depth interviews of both those who spoke publically about the research, and those who may have spoken privately, could reveal much more about the causes for stances on research by politicians. An analysis of the words used during other periods of focus on smokefree policies (such as New Zealand in 1986–1990) could provide comparisons. Surveys of politicians could provide more generalisable findings, although adequate response rates may be difficult to obtain. Studies of the use of similar research by politicians in other jurisdictions would provide valuable comparative data.

### Implications for policy and health advocates

The willingness of MPs to adopt tobacco industry arguments in the New Zealand parliament suggests that two policy initiatives may be productive for protecting public health. The enforcement of consumer protection laws by the New Zealand government, preventing deceptive information by the tobacco industry and its allies, could reduce the likelihood of MPs repeating the misinformation. Secondly, the adoption within tobacco control efforts of an increased focus on tobacco industry behaviour could have the consequence of a public better informed about tobacco industry arguments. In turn, politicians could be less likely to use the industry's arguments in public. The options for such an increased focus on tobacco industry behaviour include effective media campaigns.

More generally, health advocates could consider using such research findings to enhance the public focus at election time on politicians and parties who use tobacco industry arguments. And, underlying all the efforts to help improve the effective uptake of research by policymakers, is the need by the health sector for an increased and highly skilled advocacy capacity. This is likely to be relevant in many jurisdictions. Health research 'stories' may need exceptional storytellers (while still informed by high quality research) in order to prevail over tobacco industry 'stories'.

## Abbreviations

IARC – International Agency for Research on Cancer

MPs – Members of Parliament

SFEAA – Smoke-free Environments Amendment Act

SHS – secondhand smoke

SIDS – sudden infant death syndrome

WHO – World Health Organization

## Competing interests

The Housing and Health Research Programme/He Kainga Oranga has had contracts with the New Zealand Ministry of Health. GT and NW have both had contracts for non-profit organisations involved in tobacco control.

## Authors' contributions

GT conceived the work, collected and analysed the data and helped write the article. NW and PHC advised on all stages of the work, and helped write the article.
